# Help or Hurt? The Impact of Digital Finance on the Physical Health of the Elderly in China

**DOI:** 10.3390/healthcare12131299

**Published:** 2024-06-28

**Authors:** Yaling Luo, Lei Zhou, Weike Zhang

**Affiliations:** School of Public Administration, Sichuan University, Chengdu 610041, China; luoyy@scu.edu.cn (Y.L.); zhoulei@stu.scu.edu.cn (L.Z.)

**Keywords:** digital finance, physical health of the elderly, household disposable income, liquidity constraints, medical service utilization

## Abstract

Under the backdrop of global aging, the escalating number of elderly individuals in poor health poses a growing social burden and impacts economic development and social stability. A fundamental question arises as to whether the advancements of digital finance (DF) can effectively enhance the physical health of the elderly. This study aims to investigate the impact of DF on the physical health of the elderly by utilizing data from the China Health and Retirement Longitudinal Study (CHARLS) conducted in 2013, 2015, and 2018. The results reveal a significant positive impact of DF on enhancing the physical health of the elderly. Furthermore, the study demonstrates that this impact is particularly pronounced among the elderly with higher educational attainment, stronger intergenerational links, and those residing in central cities. A mechanism analysis further reveals that DF contributes to improving the physical health of the elderly by augmenting household disposable income, alleviating liquidity constraints, and enhancing the utilization of medical services. These findings offer valuable insights for the future development of DF and the implementation of policies promoting healthy aging and active aging.

## 1. Introduction

The world is currently undergoing notable demographic shifts, characterized by the rapid acceleration of population aging in high- and upper-middle-income countries [1]. The decline in physical functions is an inevitable consequence associated with the aging process. As this process intensifies, the proportion of the elderly facing health issues will markedly increase, leading to a significant societal burden. As a result of this, both economic development and social stability may be impacted [2]. The physical health of the elderly has emerged as a prominent global concern. As the largest developing country worldwide, China is currently grappling with the pressing challenge of population aging, which imposes a substantial burden on elderly support systems. According to a bulletin issued by China’s National Bureau of Statistics, the elderly population in China aged 60 and above reached 280 million by the end of 2022, accounting for 19.8% of the country’s total population (Statistical Bulletin of the People’s Republic of China on National Economic and Social Development for 2022, https://www.stats.gov.cn/sj/zxfb/202302/t20230228_1919011.html (accessed on 25 December 2023)). This statistic indicates that China is on the verge of entering a moderately aging society. Gao et al. (2021) report that the number of elderly individuals with disabilities or semi-disabilities will approach 100 million by this mid-century [2]. The demand and expenditure on healthcare services for the elderly will undoubtedly witness a substantial increase, thereby exerting unparalleled pressure on the healthcare supply [3]. Therefore, enhancing the physical health of the elderly is an urgent and critical issue that requires further attention.

With the advancement of information technology and the widespread adoption of smart devices, digital technology is playing an increasingly vital role in promoting the physical health of aging populations in China. According to a report conducted by the Chinese Academy of Social Sciences, the amount of WeChat payments made by the elderly has exhibited continuous growth since 2017, increasing by 5227 percent in the second quarter of 2021 compared with the first quarter of 2017 (National Institute of Social Development, CASS, http://nisd.cssn.cn/ (accessed on 25 December 2023)). As an important part of digital technology, digital finance (DF) offers convenient financial services that enhance the diversity of household investments [4] and boost the likelihood and proportion of households allocating funds to risky financial assets [5]. This can potentially alleviate financing constraints faced by the elderly, increase their income, and improve their overall quality of life. Furthermore, DF eliminates time and space constraints [6] and broadens the scope of intergenerational support. Adult children can use Internet platforms to purchase digital health insurance for their parents and make appointments online, thereby facilitating easier access to medical services for the elderly. In light of this, it is becoming extremely significant to investigate the effects of DF on the physical health of the elderly.

Currently, there is extensive academic discourse surrounding the influence of DF on the residents. This discussion primarily focuses on residents’ income, consumption, and employment [7,8,9]. Firstly, regarding residents’ income, the existing literature has reached a consensus that the growth of DF has substantially enhanced residents’ income [10]. Specifically, DF facilitates income growth by encouraging productive investment [11], self-employment [7], and financial management [12]. Secondly, some studies have demonstrated that DF promotes higher levels of consumption among residents by increasing income [13], popularizing mobile payment methods [13], providing online credit [8], and offering Internet financial products [8]. Additionally, relevant studies have indicated that DF incentivizes individuals to modify their consumption habits, adopt green consumption practices [14], and upgrade household consumption [15]. In terms of consumption types, DF has primarily contributed to the rise in household recurrent expenses [16], including food, healthcare, education, and clothing, whereas the impact on non-recurrent expenditures has been less significant [8]. Finally, some studies have indicated that DF has an overall positive effect on employment [9]. However, the impact of DF varies across industries. Specifically, DF has a greater impact on increasing labor demand in manufacturing, technology-intensive, small-city, and unlisted firms by addressing their financing constraints and operating costs [17]. Furthermore, the implementation of DF has resulted in a substantial decrease in employment within the financial industry, resulting in the emergence of skilled unemployment [18]. Nevertheless, the existing research has somewhat overlooked the circumstances of older people as a collective in the context of the advancement and dissemination of DF. There is a paucity of the literature examining the impact of DF on the lives of older people, particularly in relation to their health. Furthermore, when examining the influence of DF on residents’ income, consumption, and employment, existing studies tend to adopt a macro-level analysis, while comparatively few studies investigate micro-mechanisms. Moreover, there is a paucity of research investigating the micro-mechanisms underlying the impact of DF on the health of the elderly. Nevertheless, with the growing prevalence of DF, it is imperative to examine its influence on the elderly.

The present study utilizes data from the China Health and Retirement Longitudinal Study (CHARLS) conducted in 2013, 2015, and 2018, as well as city-level macrodata, to investigate the impact of DF on the physical health of the elderly. The conclusions of this paper are as follows. (1) The findings suggest a positive association between the development of DF and the physical health of the elderly. These findings remain robust even after conducting various robustness tests, such as replacing variables, adding potentially omitted variables, adjusting samples, and employing the instrumental variable (IV) method. (2) A further analysis reveals that the usage depth and digitization degree of DF have a notably positive impact on the physical health of the elderly, whereas the coverage breadth of DF has no significant impact. Furthermore, the beneficial impact of DF on the physical health of the elderly is mainly observed among elderly with a higher level of education, stronger intergenerational links, and those located in central cities. (3) The underlying mechanisms indicate that DF improves the physical health of the elderly primarily by increasing household disposable income, alleviating liquidity constraints, and enhancing medical service utilization.

The study makes several significant contributions. Firstly, it enriches the existing research on the effects of DF. The existing literature has primarily focused on residents’ income, consumption, and employment; this study specifically examines the impact of DF on the physical health of the elderly, thus complementing the related literature on this topic. Secondly, this study complements the research on the factors influencing the physical health of the elderly. Although limited literature has explored the impact of DF on individuals’ health from a certain aspect, such as mobile payment, no comprehensive research has been found to examine how DF affects the physical health of the elderly. Lastly, this study investigates the underlying mechanisms by considering household disposable incomes, liquidity constraints, and medical service utilization. By doing so, this study not only expands the understanding of how to improve the physical health of the elderly but also offers valuable insights for China to effectively promote the policy of active population aging.

The remainder of this study is organized as follows: The second part presents the theoretical analysis and research hypotheses. The third part outlines the data, variables, and model. The fourth part discusses the empirical results. The fifth part delves into the underlying mechanisms. Finally, the study concludes with a summary and policy implications.

## 2. Theoretical Analysis and Hypothesis Development

### 2.1. DF and Physical Health of the Elderly

China’s National Health Commission defines the health of the elderly as a state in which individuals aged 60 years or older achieve coordination and harmony among their physical, psychological, and social aspects, enabling them to live independently or with a basic level of independence (the standard for healthy Chinese older adults, http://zs.kaipuyun.cn/s (accessed on 28 December 2023)). The primary focus of this study is to analyze the influence of DF on the physical health of the elderly. Physical health in this context refers to the overall well-being and functional integrity of the elderly body. Currently, research on elderly health predominantly focuses on identifying factors that influence their well-being. Previous studies have identified four broad categories of primary factors that affect the physical health of the elderly: demographic factors [2], socio-economic factors [19], health behaviors [20], and social relationships [19].

As one of the socio-economic factors, DF is a financial service model that can affect the lifestyle and physical health of the elderly. DF utilizes mobile communication and Internet technology to provide financing, investment, wealth management, and other related services, using contemporary digital information as a medium [21]. It incorporates a wide range of financial products, services, software, and next-generation customer engagement models [22]. In recent years, China’s DF has experienced rapid growth, covering areas such as digital payment, financial management, online insurance, digital currency, and Internet-based financing [23].

In traditional financial markets, the elderly face significant challenges in accessing financial services, resulting in financial exclusion [24]. On one hand, factors such as poor financial literacy, singular economic resources, memory loss, and mobility issues contribute to a decrease in access among the elderly. On the other hand, the availability of financial products specifically tailored to meet the needs of the elderly is limited and inadequate. This lack of personalized options fails to adequately address the financial requirements of the elderly. The consequences of financial exclusion extend beyond financial matters and can negatively impact both household asset allocation efficiency and the overall quality of life for the elderly, ultimately affecting their physical health [25,26].

However, DF has the potential to alleviate financial exclusion and improve the physical health of the elderly. Firstly, DF promotes inclusivity by providing financial services to underserved areas and users through Internet technologies [10]. This approach ensures equal opportunities and enables the elderly to benefit from the advancements in DF. Secondly, DF can mitigate information constraints by reducing information asymmetry in the financial market through technologically advanced methods such as big data, blockchain, and AI [27]. Consequently, this expands the elderly’s access to information, improves their financial literacy, optimizes investment decisions, and reduces the likelihood that they encounter financial exclusion. Thirdly, DF offers a wide range of services. Considering the cost of risk assessment, traditional financial institutions predominantly focus on individuals with substantial financial needs and good credit qualifications [28]. In contrast, digital financial institutions, with the help of information technology, can more accurately assess the credit risks of the elderly at a lower cost, thereby increasing the availability of financial products tailored to their needs. Finally, DF benefits from economies of scale. By leveraging the Internet, DF overcomes limitations of time and space [10], resulting in reduced marginal expenses and increased efficiency. As a result, DF can provide accessible and efficient financial services, effectively addressing the financial exclusion of the elderly and ultimately improving their physical health.

Therefore, this study proposes the following hypothesis:

**H1:**  *DF can improve the physical health of the elderly.*

### 2.2. DF, Household Disposable Income, and Physical Health of Elderly

DF plays a crucial role in fostering investment in the health of the elderly through enhancing their income status and that of their family. According to the health capital demand theory proposed by Grossman (1972) [29], higher income levels lead to increased investment in health. DF facilitates investment and financial management for the elderly and their households. By integrating information technology, DF can broaden its reach to include a greater number of elderly individuals and their households [30], lessening their dependence on the physical branches of financial institutions [31]. Additionally, DF has expanded the depth of financial services by leveraging big data to monitor user requirements. This enables financial institutions to offer diversified financial products and online transaction channels to households [32], potentially increasing their property income. These improvements in the coverage and depth of financial services have reduced the costs associated with accessing them, allowing individuals at the lower end of the income distribution to obtain them for a reasonable cost [31]. As a result, this has expedited capital accumulation and bolstered society’s overall income level.

Furthermore, the development of DF can also enhance the financial literacy of seniors and their families. DF significantly enhances the dissemination of financial information, facilitating communication between traditional commercial banks and households, regardless of the geographical constraints imposed by bank branches [33]. By relying on digital platforms, DF eliminates geographical restrictions and provides elderly households with abundant financial information, thus enhancing their financial literacy [33]. This, in turn, enables elderly households to effectively leverage the resource allocation and risk mitigation functions of DF, overcoming geographical and price-based barriers to financial inclusion, while also diversifying their income streams [31]. Simultaneously, it also empowers the elderly and their households to make informed investment decisions and enhance the efficiency of their investment portfolios [34].

Finally, DF can enhance accessibility to professional investment advice for the elderly and their families. The investment advisory services offered by traditional commercial banks are typically expensive and inefficient, which discourages many households from utilizing them [34]. However, the emergence of DF has led to a significant increase in the adoption of robo-advisors that leverage big data and artificial intelligence [35,36]. Through online platforms, the elderly and their households can now access faster and more cost-effective consultation services from robo-advisors, thereby reducing irrational emotions caused by limited information [35]. This, in turn, improves the efficiency of investments, ultimately increases income, and enhances healthcare provision for the elderly. In summary, DF significantly contributes to elevating the income levels of the elderly and their households, ensuring sufficient funds for healthcare expenditure, and enhancing the physical well-being of the elderly.

Therefore, this study proposes the following hypothesis:

**H2:**  *DF may improve the physical health of the elderly by increasing household disposable income.*

### 2.3. DF, Liquidity Constraints, and Physical Health of Elderly

Alleviating liquidity constraints is another critical channel through which DF affects the healthy investing of the elderly. Liquidity constraints constitute a significant determinant impacting health investment [37,38]. The previous literature demonstrates that such constraints can impede or even obstruct one’s timely access to medical treatment for individuals in need [26], thereby exacerbating their health conditions. Moreover, stringent liquidity constraints exert heightened pressure on the elderly, potentially leading to delayed retirement and reduced leisure or exercise time dedicated to work [39], which is detrimental to the physical well-being of the elderly [40]. The advancement of DF has the potential to greatly reduce the liquidity constraints faced by the elderly and their households.

Firstly, DF can mitigate information asymmetry in credit markets. In traditional credit markets, limited lender information necessitates financial institutions to rely on collateral as a means to address moral hazard and adverse selection in credit transactions [41], resulting in the exclusion of potential borrowers. However, DF effectively facilitates the integration of digital technology and financial institutions, enabling them to acquire lender information at a reduced cost, thereby diminishing risk control expenses and alleviating credit exclusion [42]. Specifically, DF has established a range of “scenarios”, such as Taobao and WeChat, that transcend temporal and spatial limitations to connect customers’ mobile devices and gather vast amounts of mobile data [43]. Through the accumulation of this data, the marginal cost of developing relevant financial services is reduced, effectively addressing issues related to diseconomies of scale while simultaneously lowering risk control costs [44]. Additionally, digital financial institutions rely on advanced information processing techniques such as big data to optimize and analyze this mobile data, which enables effective risk assessment and loan evaluation [44], thereby facilitating the provision of credit support to a larger number of elderly individuals and their households. Ultimately, this contributes to mitigating their liquidity constraints.

The second benefit of DF is that it enhances credit accessibility for the elderly and their households. On one hand, DF has revolutionized the accessibility and convenience of financial services by leveraging disruptive technologies like AI, blockchain, cloud computation, and big data [45]. This advancement surpasses the constraints posed by physical branches in the traditional finance industry [31]. On the other hand, the loan application process has been simplified to a user-friendly online terminal experience, catering especially to elderly individuals with limited financial knowledge [46].

Finally, DF can help the elderly and their households better achieve risk smoothing. Before the proliferation of mobile payments, the majority of households used China Post or bank transfers as a means for remittances, which constituted a conventional process that entailed relatively high costs and consumed significant time [47]. With the advancement and widespread adoption of mobile payment, individuals now have the capability to transfer funds to others in real time with minimal transaction costs [48]. As a result, the elderly facing heightened liquidity constraints can benefit from receiving instantaneous transfers from their relatives and friends at reduced expenses, thereby enhancing their risk mitigation capabilities. In addition, mobile payment in China has evolved into an electronic wallet that can generate liquidity and savings effects [49]. For instance, Yu e‘ Bao, the largest online money market mutual fund provided by Alipay, enjoys widespread acceptance [50]. Consequently, the elderly and their households can establish precautionary savings with enhanced liquidity through investments in Internet money market mutual funds. To sum up, DF significantly alleviates the liquidity constraints faced by the elderly and their households, thereby facilitating increased investment in leisure activities, physical exercise, and other related areas. Ultimately, this leads to improved health outcomes for the elderly.

Therefore, this study proposes the following hypothesis:

**H3:**  *DF may improve the physical health of the elderly by alleviating liquidity constraints.*

### 2.4. DF, Medical Service Utilization, and Physical Health of Elderly

DF plays a vital role in the health investments of the elderly by increasing medical service utilization. The elderly population is particularly susceptible to fluctuations in medical service utilization [51,52], and the inadequate use of medical services can result in unmet health needs among this demographic, leading to the avoidable deterioration of their health condition [51]. Adhvaryu and Nyshadham (2015) have also demonstrated that actively seeking formal medical services can significantly improve health outcomes for the elderly [53]. DF has the potential to improve the utilization of medical services for the elderly.

The primary advantage of DF lies in its capacity to facilitate the acquisition of health insurance for the elderly. From the perspective of the supply side, health insurance companies can utilize information technology to segment and repackage diverse insurance options, thereby enhancing the personalization and humanization of health insurance design [54]. This enables them to offer a wide range of health insurance products that cater to the needs of various customer segments [54], particularly the elderly. Additionally, DF can enhance the ability of health insurers to accurately identify customers and conduct targeted insurance marketing [55], thereby improving marketing efficiency for the elderly. From the demand side, DF improves the accessibility of health insurance for the elderly and also reduces the cost of purchasing health insurance. On one hand, the Internet platform effectively reduces search costs for the elderly and their households [55,56] while also providing convenient access to compare comprehensive information and prices of various Internet health insurance products [56]. This empowers them to meticulously select products that align with their specific needs, thereby augmenting the likelihood of making a purchase. On the other hand, the utilization of Internet-based financial services by the elderly, such as digital payments, may lead to their exposure to Internet health insurance products through the establishment of a robust network connecting various Internet financial services [57]. DF ultimately facilitates the accessibility of health insurance for the elderly, significantly mitigates the financial burden associated with medical services, and actively promotes timely healthcare-seeking behavior among this demographic.

Secondly, DF amplifies the social connectedness of the elderly and augments their social capital. Existing research suggests that social capital can have a significant impact on physical and mental health [58]. The digital platforms in China, such as WeChat and Alipay, not only provide digital financial services but also offer virtual social functionalities [59,60]. The adoption of big data-driven digital payment systems by the elderly amplifies their interpersonal connectivity, augmenting the scope and strength of their social relationships [61]. This broadening of social networks offers them supplementary avenues for acquiring health-related knowledge and seeking medical advice [62]. Moreover, mobile payment enhances the connectivity of the elderly with their families, hospitals, and other entities, thereby providing them with increased opportunities and resources [48]. This facilitates a reduction in transaction costs associated with medical services. For instance, DF enables remote registration and online medical consultations, thereby improving the efficiency and convenience of medical services for the elderly [61]. Consequently, this can ultimately enhance the utilization of medical services by the elderly. In conclusion, DF holds the potential to enhance the utilization of medical services among the elderly by facilitating health insurance purchases and fostering the expansion of social capital. This can tackle the phenomenon of ‘untreated illnesses’ by enabling timely disease prevention and intervention measures for the elderly population [63], thus contributing significantly to their overall health maintenance and improvement.

Therefore, this study proposes the following hypothesis:

**H4:**  *DF may improve the physical health of the elderly by increasing medical service utilization.*

**Figure 1:**
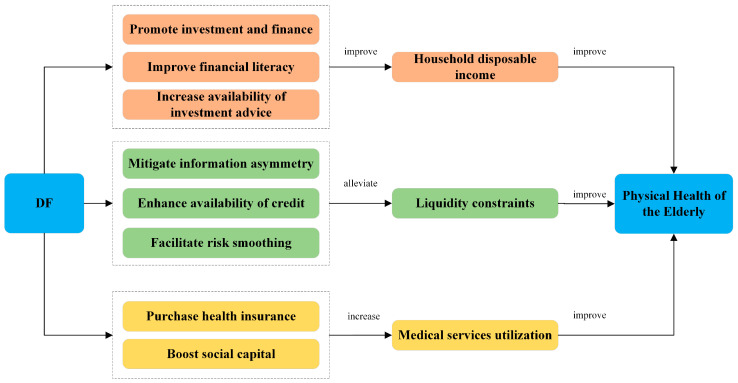
Theoretical research framework.

Figure 1 summarizes the theoretical analysis framework of the impact of DF on the physical health of the elderly. This theoretical framework outlines three principal mechanisms through which DF impacts the physical health of older adults and details the various subfields where these mechanisms operate. Specifically, building upon a thorough review and analysis of the literature, this paper posits that DF may primarily impact the physical health of the elderly by influencing household disposable income, liquidity constraints, and medical service utilization. Furthermore, this study delves into domains closely related to DF and the daily lives of the elderly, integrating current research to thoroughly examine how DF impacts these three potential mechanisms. This analysis elucidates the role of DF across various sub-domains, detailing its effects on the health outcomes of older adults.

## 3. Methods

### 3.1. Data

This study utilizes both urban and individual data to investigate the impact of DF on the physical health of the elderly in China. The individual data are derived from the CHARLS from 2013 to 2018 (the release of the CHARLS 2020 data has been postponed due to the outbreak of COVID-19, and as of writing, the most recent data have not been made available to the public), which is equivalent to the Health and Retirement Study conducted by the University of Michigan in the United States. The CHARLS, conducted by the National Development Research Institute of Peking University, encompasses 150 county-level units, 450 village-level units, and 17,000 individuals residing in approximately 10,000 households. Its objective was to gather high-quality microdata that represent typical Chinese midlife and elderly households, as well as individuals aged 45 and above. The city-level data were obtained from the Peking University Digital Financial Inclusion Index and statistical yearbook in corresponding years. The former, a collaborative effort between the Digital Finance Research Center of Peking University and Ant Financial Services Group, assessed the development of China’s digital finance industry across 31 provinces and cities, 337 cities above the prefecture level, and 1754 counties by evaluating the sector’s depth of usage, breadth of coverage, and level of digitization. It serves as a valuable resource for this study.

The data in this study have been processed in accordance with the research requirements and data characteristics. Firstly, this study matches the CHARLS data from 2013, 2015, and 2018 with the corresponding city data. Secondly, this study excludes the samples with ages below 60, along with samples that have only one year of data or are missing critical factors. Thirdly, this study winsorizes all continuous variables at the 1st and 99th percentiles. Finally, a total of 11,150 samples have been retained for analysis.

### 3.2. Variables

#### 3.2.1. Explained Variable

The explained variable of this study is the physical health of the elderly. Following the previous research of Chen et al. (2022), this study employs the IADL scores as proxy measures for assessing the physical health of the elderly [64]. (The IADL includes shopping, cooking, eating, bathing, dressing, climbing stairs, housekeeping, managing money, taking medication, making phone calls, going to the toilet, and one’s control of urination and defecation.) Specifically, the CHARLS questionnaire categorizes each IADL into four categories: “no difficulty”, “difficult but still able to complete”, “difficult and needs help”, and “unable to complete”. Each category is numerically assigned a value of 4, 3, 2, or 1, respectively. The resulting scores for the physical health of the elderly range from 12 to 48. A higher score indicates a better health status for the elderly.

#### 3.2.2. Explanatory Variable

The explanatory variable in this study is DF, which is measured using the DFI index proposed by the Digital Finance Research Center of Peking University. The DFI index comprises three indicators: coverage breadth, usage depth, and digitization degree. Coverage breadth is determined by the degree of DFI, which includes the number of Alipay users, the proportion of Alipay-linked card users, and the number of account-bound cards. Usage depth reflects the multiplicity of financial services utilized, such as payments, credit, insurance, and investment. The digitization degree primarily takes into account factors like liquidity, affordability, credit, convenience, and other factors, which reflect the low cost and universality of financial services. Additionally, this paper conducts a comprehensive analysis of the core explanatory variables, namely coverage breadth, usage depth, and digitization degree, to account for heterogeneity.

#### 3.2.3. Control Variables

The study incorporates control variables at both individual and city levels. The individual-level variables include age, marital status, income, social health insurance, and commercial health insurance. The city-level variables encompass economic growth, medical condition, traditional financial size, and air quality.

Table 1 presents the descriptive statistics for the main variables, including the full sample, between-group comparisons, and within-group analyses. The physical health of the elderly, which is measured using the IADL scores of individuals, has a mean of 44.063 and a standard deviation of 5.414. The scores range from 12 to 48, with a minimum value of 17 and a maximum value of 48. The findings suggest that the physical well-being of the elderly is generally satisfactory, although significant variations exist among individuals. The variable DF, which quantifies the degree of digital financial advancement in a city, has a mean of 180.258, a minimum of 102.240, and a maximum of 281.971. The results indicate that the DF in the sample cities has achieved a certain level of development. However, the extent of development is uneven across cities. Additionally, the T-bar value of 2.399 indicates that this study’s dataset is unbalanced, attributable to missing data from CHARLS. This could potentially bias the results. To address this issue, the study employs fixed effects models and IV methods. Furthermore, the within-group descriptive statistics of the variables have been decentralized, which helps control for unobservable individual heterogeneity, thereby enhancing the reliability of the analyses.

Table 2 presents the correlation coefficient matrix of all independent variables. It can be observed that there is a strong correlation among some variables, which indicates the potential presence of a multicollinearity problem. To further investigate this, this paper employs the variance inflation factor (VIF) to assess the extent of multicollinearity among the variables. The maximum value of the VIF is 2.55, while the average value is 1.52, which is considerably less than 10. This indicates that the variables used in this paper do not exhibit multicollinearity.

### 3.3. Model

This study constructs the following model to investigate the impact of DF on the physical health of the elderly in China:

(1) $$Y_{ijt}=\beta_{0}+\beta DF_{jt}+\lambda \times Controls+\theta_{i}+\lambda_{j}+\sigma_{t}+\varepsilon_{ijt}$$

where *Y* is the explained variable of the physical health of the elderly. *DF* represents the explanatory variable of DF. Its corresponding coefficient *β* is the focus of this paper, reflecting the effect of DF on the physical health of the elderly, and the value of *β* is expected to be positive. *Controls* denote a collection of control variables, including individual-level and city-level characteristics. $\theta_{i}$, $\lambda_{j}$, and σ represent individual, city, and year fixed effects, respectively. *i*, *j*, and *t* represent individual, city, and year, respectively. *β*_0_ is an intercept term, while *ε* is a random disturbance term. In this study, multilevel modeling is also a feasible analytical approach. However, due to the absence of relevant variables at the individual level and the superior ability of the fixed effects model to control for unobserved characteristics at the time level, multilevel modeling was not employed. Future studies may consider this modeling approach, provided that the data availability allows for this. Furthermore, this paper conducted a Hausman test on the fixed effects model and the random effects model. The value of the chi-square was 47.09, and the *p*-value was considerably less than 0.05, which generally indicates that the hypothesis is not valid that asserts that the individual effects assumed in the random effects model are not correlated with the explanatory variables. Consequently, the fixed effects model is more appropriate for this dataset.

## 4. Results and Discussion

### 4.1. Baseline Results

The results of the baseline regression are presented in Table 3, where columns (1)–(3) sequentially introduce different levels of control variables to improve the accuracy of the estimates. Column (1) is devoid of any control variables, column (2) comprises variables pertaining to the characteristics of an individual, and column (3) integrates variables related to both the characteristics of an individual and a city.

The coefficients of DF in columns (1)–(3) are all significant at the 1% level. Specifically, the coefficients are 0.1048, 0.047, and 0.039, respectively. As the control variables at the individual and city levels are progressively included, the coefficients of DF gradually decrease. These findings suggest that the expansion of DF has a significant favorable effect on the physical health of the elderly, thus confirming hypothesis H1. This finding is in accordance with the findings of Zhang et al. (2022), whose empirical analysis utilizing data from the 2017 China Household Finance Survey demonstrates that mobile payments have the potential to enhance citizens’ well-being by streamlining health insurance purchases and enabling leisure expenditure [65]. However, it is important to note that Zhang et al. (2022) focus exclusively on mobile payment services within DF and do not comprehensively examine DF specifically to investigate its impact on the physical health of the elderly as a demographic group [65].

A possible explanation for this considerable impact of DF on the physical health of the elderly is that DF based on an Internet platform provides low-cost and accessible financial services, mitigates information asymmetries in financial markets, and optimizes the financial market environment. This program enhances the financial accessibility of elderly individuals, increases their income, and alleviates liquidity constraints, which in turn helps them invest in their health, cope better with health shocks, and ultimately achieve improved health status.

### 4.2. Robustness Tests

#### 4.2.1. Alternative Measure of the Physical Health of the Elderly

In order to strengthen the credibility of the baseline estimation, this study employs the chronic disease indicator as a measure of the physical health of the elderly to conduct a robustness test. Drawing on relevant inquiries in the CHARLS dataset, this study constructs a chronic disease indicator, focusing on three commonly occurring chronic illnesses that are more prevalent in older age: hypertension, stroke, and dementia. Each respondent is assigned a point for each illness, with lower scores indicating a lower risk of chronic disease and better health of the elderly. The results of this estimation are presented in column (1) of Table 4. It is evident that the estimated coefficient of DF is significantly negative at the 1% level, thereby confirming the robustness of the aforementioned finding.

#### 4.2.2. Alternative Measure of DF

The baseline regression assesses the development level of DF by utilizing the DFI index at the city level. To ensure the soundness of the baseline regression, this section conducts a robustness test using the DFI index at the province level as a proxy measure of DF. The estimation results, presented in column (2) of Table 4, reveal a significantly positive coefficient for DF at a 1% level of significance, thereby confirming its reliability in validating the core findings of this study.

#### 4.2.3. Controlling Additional Variables

In the previous baseline regression, this study accounts for various characteristic variables of individuals and cities. However, it is essential to acknowledge the potential presence of an omitted variable, specifically the mobile phone penetration rate. Previous studies have indicated that the utilization of smartphones and the Internet has a considerable impact on the health of the elderly population [66]. Moreover, it has been observed that as the mobile phone penetration rate increases, there is an increased likelihood for the elderly to be exposed to smartphones and the Internet. Thus, the current study includes mobile phone penetration rate as an additional control variable to conduct a robustness test. The estimation results, presented in column (3) of Table 4, reveal that even after accounting for mobile phone penetration rate, the coefficient of DF remains significantly positive at the 1% level, and its value does not undergo significant changes, further reinforcing the credibility of the baseline estimation.

#### 4.2.4. Excluding Samples

In order to enhance the robustness of the aforementioned findings, this study excludes samples from first-tier cities (Beijing, Shanghai, Guangzhou, and Shenzhen), Hangzhou city, and provinces with high DFI (Jiangsu, Zhejiang, and Fujian), respectively. Specifically, China’s first-tier cities boast significant economic progress and superior public service delivery [67]. Additionally, Hangzhou is renowned as a hub for digital economy development, boasting one of the highest concentrations of digital economy businesses in China [68]. It consistently ranked first in DFI during the sampled years of 2013, 2015, and 2018. Furthermore, the Jiangsu, Zhejiang, and Fujian Provinces demonstrate a much higher-than-average DFI. To address the potential influence of specific cities and provinces, this study eliminates data from the first-tier cities, Hangzhou city, and provinces with high DFI for the regression analysis, respectively. The findings are presented in columns (1)–(3) of Table 5. After excluding these samples, the estimated coefficients of DF remain significantly positive at the 1% level. These outcomes align with the findings obtained from the baseline regression, once again confirming the reliability of the core findings in our study.

#### 4.2.5. IV Methods

This paper incorporates confounding control variables at both the individual and city levels in the baseline regression and also addresses the possibility of omitted variables. Nonetheless, it cannot guarantee the complete correction of endogeneity bias resulting from omitted variables, nor can it entirely eliminate the issue of reverse causality. To address these concerns, this study employs an instrumental variable (IV) method. Specifically, the average DFI index of other cities, the number of households with Internet access in the previous year, and the interaction term between the number of fixed-line telephones per 100 people in the city in 1984 and the number of households with Internet access in the previous year are employed as the instrumental variables for DF. The estimation results derived from the IV method with a two-stage least square (2SLS) are presented in Table 6.

The results suggest that the first-stage F statistics for the three instrumental variables are greater than 10, implying the absence of weak instrumental variable issues. The Cragg–Donald Wald F-values surpass the critical value of the Stock–Yogo weak ID test at the 10% significance level (16.38), signifying the successful passing of the under-identification test. The results obtained through the IV method consistently exhibit positive and statistically significant coefficients at the 5% level or higher, aligning with the baseline results. Additionally, the estimated coefficients of DF are considerably higher compared to the baseline results, suggesting that the endogeneity problem has been partially mitigated and further confirming the robustness of our conclusions.

### 4.3. Heterogeneity Analysis

#### 4.3.1. DF Dimensions

The DFI index comprises three indicators: coverage breadth, usage depth, and digitization degree, which represent different dimensions of DF development. Previous studies have demonstrated that these distinct dimensions of DF have diverse impacts on economic and social aspects [69]. Therefore, it is imperative to investigate whether the different dimensions of DF have heterogeneous effects on the physical health of the elderly. To address this issue, the three primary indices of DFI have been selected as explanatory variables for regression analysis.

The results presented in Table 7 indicate that the coefficient for coverage breadth is not statistically significant at the traditional levels (*p* > 0.10). However, the coefficients for usage depth and digitization degree are significantly positive at a level of 1% or 5%. These findings highlight the varying impacts of diverse dimensions of DF on the physical health of the elderly. Specifically, the usage depth and digitization degree of DF have a significant positive effect on the physical health of the elderly, whereas the coverage breadth of DF does not demonstrate a significant impact. This lack of significance may be attributed to the limited scope of the coverage breadth indicator, which solely considers the number of Alipay users in the region. Merely having an increased number of users does not necessarily imply that the elderly demographic has actively engaged with DF services or derived health benefits from them. Thus, the coverage breadth of DF has no notable impact on the physical health of the elderly.

On the contrary, the usage depth is intended to gauge the actual frequency of DF service utilization in a particular area, thereby providing a more comprehensive assessment of DF progress in that area. It is also noteworthy that the investigated DF services, such as health insurance, are closely intertwined with the physical health of the elderly. Moreover, the digitization degree prioritizes the efficiency and convenience of regional DF services. By enhancing the accessibility and performance of DF services, it will be possible to lower the threshold for the elderly to access and utilize these services [70]. Consequently, this can help alleviate the financial exclusion experienced by the elderly and simplify their ability to benefit from the health advantages offered by DF services.

#### 4.3.2. Intergenerational Links

DF services, which depend on Internet platforms, may face obstacles in benefiting the elderly due to the digital divide [71]. Previous studies have shown that when the children of the elderly provide support and assistance with digital technology, it helps bridge the digital divide and enables them to access and utilize the Internet [72]. The availability of intergenerational digital support for the elderly is closely associated with the strength of their connections with their children [73]. The stronger the intergenerational links, the more likely the elderly are to obtain cognitive influence and support from their children regarding Internet use. Consequently, it is essential to investigate whether the promotional impact of DF on the health of the elderly differs as a function of the strength of intergenerational relationships. Five indicators are employed in this study to measure the intensity of intergenerational links: cohabitation with children, not cohabitation but in the same city, not cohabitation and not in the same city, not cohabitation but in weekly contact, and not cohabitation and no weekly contact.

The results demonstrate a significant positive coefficient of DF on the physical health of the elderly when they cohabit with their children, do not cohabit but are in the same city, or are in weekly contact. Nevertheless, the elderly who do not cohabit with their children and do not live in the same city, or do not have weekly contact have an insignificant coefficient on DF. These findings lend credence to the notion that the efficacy of DF on the physical health of the elderly appears to be differentially dependent on the strength of intergenerational links. This can be attributed to the support of digital resources across generations. When faced with digital technology, the elderly experience obstacles and challenges related to their cognitive and physical capabilities [74]. The preferred approach for them is to seek assistance from family members [72]. Seniors who cohabit with their children, do not cohabit but are in the same city, or are in weekly contact are more inclined to receive digital literacy training, operating instructions, and smart devices from their children. As a result, they are more likely to benefit from income and credit support from DF services to enhance their health [75] (Table 8).

#### 4.3.3. Educational Level

The existing literature suggests that individuals with higher levels of education are more likely to benefit from the dividend of the digital economy to a greater extent, both in terms of employment opportunities [76] and consumption patterns [77]. DF, as a crucial component of the digital economy, may also be affected by the level of education in terms of its potential benefits for the physical health of the elderly. Given this, this study aims to examine the varied impacts of DF on the physical health of the elderly, considering the educational background of the elderly. This study examines the educational attainment of the elderly by analyzing their years of formal education. The sample is divided into low and high educational levels depending on the median number of years of schooling for the elderly. The corresponding results are displayed in columns (1)–(2) of Table 9.

The findings demonstrate a significantly positive estimated coefficient (*p* < 0.01) for the impact of DF on the physical health of the elderly with higher levels of education. In contrast, the coefficient of DF relating to the elderly with lower levels of education is insignificant at the traditional levels. These results indicate that the effect of DF on the physical health of the elderly varies depending on their educational background. One possible explanation for this disparity is the influence of educational levels on the financial literacy and financial perceptions of the elderly [78], which in turn affects their financial decisions. The elderly with higher levels of education are better positioned to access information and acquire the necessary financial knowledge for making optimal financial decisions [79,80]. Furthermore, the elderly with higher-level educational backgrounds are more open to innovative financial service models that depend on online platforms, leading them to engage more frequently and extensively in DF [81]. Consequently, they are more inclined to benefit from digital technology.

#### 4.3.4. Urban–Rural

Urban areas in China outclass rural areas in multiple dimensions of development, such as income, education, healthcare, and public services [82]. The disparity between urban and rural areas poses a significant challenge to achieving balanced economic development in China. Consequently, it is crucial to examine the distinct impact of DF on the physical health of the elderly in both urban and rural areas. To investigate this matter, this study divides the sample into urban and rural areas, and the results are presented in columns (3)–(4) of Table 9.

The results demonstrate that the coefficient for DF is significantly positive at the 1% level in urban areas whereas it lacks significance in rural areas. These findings suggest that the development of DF can significantly enhance the physical health of the elderly in urban areas but it does not have a significant impact on the physical health of the elderly in rural areas. One possible explanation for this disparity is the insufficient digital infrastructure in rural areas [83], which hinders the growth of the digital finance industry. Specifically, the elderly in rural areas typically lack intergenerational support due to the fact that most young people leave to work outside their hometowns [84]. Furthermore, the digital divide presents a challenge to the widespread adoption and utilization of DF services among the rural elderly [85]. Consequently, the development of DF has limited potential for enhancing the physical health of the elderly in rural areas compared to their urban counterparts.

#### 4.3.5. Region

The classification of China by the National Bureau of Statistics into three regions, namely Eastern, Central, and Western China, reveals considerable disparities in economic growth and medical services persist among these regions [86,87]. Consequently, the impact of DF development on the physical health of the elderly may vary across these regions. To address this regional variation, the sample in this study is divided into three groups: eastern, central, and western. The regression results for each group are presented in columns (1)–(3) of Table 10.

The findings reveal a significantly positive DF coefficient in the central region at traditional statistical levels. However, the DF coefficients for the eastern and western regions do not exhibit statistical significance. This evidence implies that the advancement of DF has enhanced the physical well-being of the elderly solely in Central China but it has not impacted the physical health of the elderly in the eastern and western regions. This variability can be attributed to the varying stages of development within the digital finance industry across various regions. The digital finance industry in the eastern region was initiated early and reached a high level [88], which likely resulted in observable health benefits prior to the sample period. As a result, the elderly in this region may have become less sensitive to shocks related to the development of DF during the sample period. In contrast, the DF in the western region started later and developed at a lower level [89], which may have presented infrastructural challenges that hindered potential health benefits. Consequently, the health-promoting effects of the western region’s DF have not yet been observed. It should be noted that the central region is experiencing thriving growth in the digital finance industry, accompanied by ongoing infrastructure improvements [88]. As a consequence, the stimulating effect of DF on the physical health of the elderly is quite significant.

## 5. Underlying Mechanism

### 5.1. Household Disposable Income

Existing research has established that the elderly with higher household incomes experience better living conditions and are more likely to invest in their health, leading to improved overall health outcomes [89,90]. DF plays an indispensable role in facilitating the investment of money by elderly households, thereby increasing their access to income. By offering convenient financial products through the Internet, DF simplifies the process of investing for elderly households. Consequently, DF has a favorable potential impact on the physical health of elderly individuals by improving their disposable income. This study aims to examine whether DF enhances the physical health of the elderly by bolstering the disposable income of their households. To investigate this relationship, per capita household income (measured in ten thousand CNY) is utilized as a surrogate indicator for the disposable income of elderly households. The selection of this proxy variable is based on the definition of “household members” in the CHARLS survey, which includes cohabitating family members with shared earnings and expenses. Moreover, using per capita household income as a representation of personal income is particularly relevant for older adults due to its practical significance.

The findings of this research suggest that the coefficient for DF is 0.013 and statistically significant at the 1% level. These results suggest that the expansion of DF is essential to enhance the income of elderly households and improve their physical health, thereby supporting hypothesis H2. On one hand, DF relies on Internet digital platforms to offer a plethora of accessible and convenient financial products [70], leading to reduced investment costs for the elderly and motivating their engagement in financial management. On the other hand, the growth and spread of DF have enabled the broad circulation of financial knowledge and information across financial markets, leading to enhanced financial literacy [8] and better decision-making regarding wealth management among elderly households. Consequently, the income of the elderly and their households has risen, ultimately contributing to the improvement of the physical health of the elderly.

### 5.2. Liquidity Constraints

Liquidity constraints can exert a significant impact on the well-being of the elderly [26]. Severe liquidity constraints can generate heightened psychological stress in the elderly, while also restricting their willingness and ability to invest in their long-term health, ultimately resulting in a decline in their physical and mental welfare. The emergence of DF holds the potential to decrease financial exclusion among the elderly by lowering credit thresholds and enhancing service efficiency [70], thereby alleviating their liquidity constraints. Thus, DF has the capacity to impact the physical health of the elderly by relieving their liquidity constraints. This study aims to assess this potential mechanism by measuring the liquidity constraints experienced by the elderly in terms of household liquid assets (measured in ten thousand CNY). The results are presented in column (2) of Table 11.

The findings reveal that the estimated coefficient of DF is positive, which is statistically significant at the 10% level. These results provide evidence supporting hypothesis H3, indicating that DF plays a vital role in mitigating liquidity constraints among the elderly and ultimately improving their physical health. Firstly, DF, with its inclusiveness, optimizes the financial environment and improves financing efficiency, thereby lowering the threshold and cost of formal finance. This provides easier credit support for the elderly, thereby alleviating liquidity constraints to some extent. For another thing, digital financial institutions can utilize big data and artificial intelligence to leverage the fragmented information generated by seniors when they use DF services [43,44]. This can help to accurately pinpoint their financial risks and forecast their creditworthiness, thereby diminishing information asymmetry, easing their credit constraints, and ultimately alleviating their liquidity constraints and enhancing the physical health of the elderly.

### 5.3. Medical Service Utilization

Previous research has indicated a strong correlation between the utilization of medical services and an individual’s overall health [91,92]. By increasing the utilization of medical services, individuals can effectively minimize instances of untreated medical conditions and promptly seek preventive and interventional measures for diseases [63]. This proactive approach can prevent minor health issues from escalating into major ones, ultimately leading to an improvement in overall health and its sustained maintenance. In this context, DF offers microfinance services to assist elderly households in managing health-related emergencies. Moreover, the web-based health insurance program of DF broadens the range of health risks covered for the elderly while bolstering the healthcare spending capabilities for elderly households. Thus, DF has the potential to positively impact healthcare utilization among the elderly, specifically in the domain of health emergencies. To investigate this potential mechanism, this study assesses the medical service utilization among the elderly by analyzing household healthcare consumption over the previous year (measured in ten thousand CNY). This proxy variable is chosen as consumption in the CHARLS data aggregated at the household level, and the consumption behavior of household members is influenced by similarities and economies of scale. It is crucial to acknowledge that older adults may experience diverse health concerns, with associated treatment costs that may vary. However, this study controlled for social health insurance participation and commercial health insurance participation among older adults. The differential reimbursement rates applied by health insurance providers to different illnesses serve to mitigate this concern to a certain extent [93].

The results illustrate that the estimated coefficient of DF is significant and positive at the 10% level. This confirms hypothesis H4, which demonstrates that the development of DF has a substantial impact on increasing the utilization of medical services among the elderly and consequently improves their physical health outcomes, confirming hypothesis H4. On one hand, DF facilitates financial investment and provides appropriate credit services for the elderly, alleviating their credit constraints and ensuring financial stability when faced with health risk shocks. This enables the elderly to seek timely medical attention and fully utilize available medical services. On the other hand, DF also offers multilevel Internet health insurance [56] and targeted insurance businesses that encourage the purchase of health insurance for the elderly, diversifying their health risks. Private insurance companies covering a portion of medical costs further enhance the willingness and ability of the elderly to utilize medical services, ultimately leading to improved health outcomes for the elderly.

## 6. Conclusions

In recent years, the surge in the digital economy has become a key driver of global economic development. DF, in particular, has infiltrated various aspects of people’s livelihoods, serving as a noteworthy manifestation and application of the digital economy. DF offers a reliable avenue for improving the financial well-being of the elderly and their families, thereby boosting their physical health outcomes. In light of this, this study aims to investigate the impact of DF on the physical health of the elderly and explore the underlying mechanisms. To achieve this, this study utilizes CHARLS data for the years 2013, 2015, and 2018, as well as city-level macrodata. The findings of this study reveal that the advancement of DF has a substantial positive effect on the physical health of the elderly. These results remain robust even after various tests, including substituting variables, considering potential omitted variables, adjusting samples, and employing the IV method. Furthermore, the heterogeneity analysis demonstrates that the usage depth and digitization degree of DF significantly improve the physical health of the elderly, whereas its coverage breadth does not have a significant impact. In addition, the beneficial effects of DF on the physical health of the elderly are mainly observed among those with higher levels of education, stronger intergenerational links, and those residing in central cities. Finally, our analysis suggests that the ameliorative effect of DF on the physical health of the elderly primarily operates through increasing household disposable income, alleviating liquidity constraints, and enhancing the utilization of medical services. In light of the accelerated development of DF and the growing reliance of the population on it, this paper posits that the impact of DF on the physical health of older people will be even more profound. Furthermore, COVID-19 has accelerated the penetration of DF into the daily lives of the elderly, and the impact effects examined in this paper will be further significant. Therefore, with the fast evolution of digital technology, the impact of DF on the physical health of the elderly deserves more attention.

## 7. Policy Implications

Based on the aforementioned findings, this study presents the following policy prescriptions, which may be extended to other countries: Firstly, increase the depth of participation of the elderly in DF. Government policies in each country should encourage the expansion of digital financial services, such as mobile payments, online credit, and Internet insurance, and lower the threshold for the elderly to use them by incorporating appropriate aging concepts into the design of financial service products, such as streamlining operational processes. Furthermore, it is imperative for the government and social organizations to implement regular and comprehensive digital technology training programs for the elderly. These programs should cover a wide range of skills including the use of smartphones, Internet browsing, emailing, and other digital skills. It is essential that these programs be made readily available in community centers, libraries, and activity centers specifically designed for the elderly. Such initiatives would undoubtedly enhance the ability of the elderly population to effectively utilize digital technology.

Secondly, countries and regions with underdeveloped DF should prioritize the design and implementation of robust digital infrastructure, in addition to fostering international exchange. Digital infrastructure is a precondition for DF development, so further improvements in digital infrastructure construction are necessary to promote DF development. Meanwhile, it is imperative to focus on regional coordinated development within countries, focusing on strengthening the popularization of 5G and gigabit Internet services in remote areas, so as to lay a solid foundation for the development of DF in underdeveloped areas. Concurrently, it is imperative to reinforce exchanges and collaborations with international cutting-edge technology to facilitate the introduction of international advanced technology and expertise and to enhance the technical proficiency of domestic DF.

Thirdly, it is critical to recognize the synergistic effect of DF development and geriatric education. Empirical analysis indicates that as education levels increase, the beneficial effect of DF on the physical health of the elderly becomes more significant. Therefore, it is imperative to enhance policy support for geriatric education and facilitate lifelong learning for the elderly. This will effectively address the issue of the digital divide and enable more elderly individuals to benefit from the development dividends offered by the digital economy.

Finally, the financial security of the elderly should be taken seriously by governments. Specifically, financial product supervision aimed at the elderly should be strengthened and educational financial lectures tailored to this demographic should be periodically conducted. This cohort has suboptimal financial literacy and a limited ability to prevent fraud, thereby necessitating procedural adjustments in the related legal system. It is also crucial to implore children to offer adequate support and care for their elderly relatives and assist them in utilizing DF services when necessary.

## Figures and Tables

**Table 1:** Descriptive statistics.

Variables		Mean	S.D.	Min	Max	Observations
Key variables						
Physical health of the elderly (IADL scores)	Overall	44.063	5.414	17	48	N = 11,150
	Between		4.707	17	48	n = 4648
	Within		2.808	24.063	63.730	T-bar = 2.399
DF (DFI index)	Overall	180.258	47.002	102.240	281.971	N = 11,150
	Between		24.658	121.355	256.550	n = 4648
	Within		40.523	120.174	246.904	T-bar = 2.399
Individual level variables						
Age	Overall	68.881	6.321	60	89	N = 11,150
	Between		6.095	60.5	89	n = 4648
	Within		1.942	65.882	72.215	T-bar = 2.399
Income (Yes = 1; No = 0)	Overall	0.843	0.363	0	1	N = 11,150
	Between		0.249	0	1	n = 4648
	Within		0.269	0.177	1.510	T-bar = 2.399
Marital status (Married or cohabiting = 1; Unmarried, separated, and widowed = 0)	Overall	0.805	0.396	0	1	N = 11,150
	Between		0.375	0	1	n = 4648
	Within		0.130	0.139	1.472	T-bar = 2.399
Social health insurance (Yes = 1; No = 0)	Overall	0.946	0.225	0	1	N = 11,150
	Between		0.159	0	1	n = 4648
	Within		0.162	0.280	1.613	T-bar = 2.399
Commercial health insurance (Yes = 1; No = 0)	Overall	0.012	0.109	0	1	N = 11,150
	Between		0.078	0	1	n = 4648
	Within		0.077	−0.655	0.679	T-bar = 2.399
City level variables						
Economic growth (Log of GDP per capita)	Overall	10.624	0.585	9.304	12.065	N = 11,150
	Between		0.560	9.304	12.065	n = 4648
	Within		0.173	10.180	11.011	T-bar = 2.399
Traditional financial scale (Loan balance of financial institutions/GDP)	Overall	1.015	0.516	0.335	2.987	N = 11,150
	Between		0.485	0.335	2.987	n = 4648
	Within		0.181	0.080	2.041	T-bar = 2.399
Medical condition (Beds per 10,000 people)	Overall	44.762	15.617	21.452	97.140	N = 11,150
	Between		15.237	21.452	94.600	n = 4648
	Within		3.641	28.519	60.012	T-bar = 2.399
Air quality (Log of PM_2.5_)	Overall	3.642	0.515	1.802	4.555	N = 11,150
	Between		0.486	1.802	4.512	n = 4648
	Within		0.174	3.283	4.035	T-bar = 2.399

**Table 2:** Correlation coefficient matrix.

	DF	Age	Income	Marital Status	Social Health Insurance	Commercial Health Insurance	Economic Growth	Traditional Financial Scale	Medical Condition	Air Quality
DF	1									
Age	0.259 ***	1								
Income	0.012	0.039 ***	1							
Marital status	−0.067 ***	−0.262 ***	0.020 **	1						
Social health insurance	0	−0.041 ***	0.144 ***	0.050 ***	1					
Commercial health insurance	0.015	−0.017 *	−0.011	−0.012	−0.050 ***	1				
Economic growth	0.560 ***	0.114 ***	−0.005	−0.021 **	−0.021 **	0.034 ***	1			
Traditional financial scale	0.469 ***	0.098 ***	−0.014	−0.027 ***	−0.008	0.001	0.411 ***	1		
Medical condition	0.344 ***	0.041 ***	−0.020 **	−0.004	−0.021 **	0.036 ***	0.663 ***	0.614 ***	1	
Air quality	−0.271 ***	−0.063 ***	0.033 ***	0.022 **	0.022 **	0.010	−0.093 ***	−0.189 **	−0.136 **	1

*** *p* < 0.01, ** *p* < 0.05, and * *p* < 0.10. Robust standard errors are in parentheses.

**Table 3:** Baseline regression results.

Variables	Explained Variable: Physical Health of the Elderly		
	(1)	(2)	(3)
DF	0.048 ***	0.047 ***	0.039 ***
	(0.012)	(0.012)	(0.013)
Age		0.726 ***	0.727 ***
		(0.265)	(0.264)
Income		0.133	0.149
		(0.135)	(0.135)
Marital status		0.576 **	0.565 **
		(0.286)	(0.286)
Social health insurance		0.127	0.107
		(0.222)	(0.221)
Commercial health insurance		0.350	0.344
		(0.491)	(0.490)
Economic growth			0.524
			(0.406)
Traditional financial scale			−0.144
			(0.313)
Medical condition			0.039 ***
			(0.012)
Air quality			0.318
			(0.467)
Constant	35.479 ***	−15.168	−22.088
	(2.190)	(18.329)	(18.732)
Individual FE	√	√	√
City FE	√	√	√
Year FE	√	√	√
Observations	11,150	11,150	11,150
Adj_R2	0.571	0.572	0.572

*** *p* < 0.01, ** *p* < 0.05. Robust standard errors are in parentheses.

**Table 4:** Robustness test of alternative variables and additional variables.

Variables	Alternative Measure of Physical Health of the Elderly	Alternative Measure of DF	Controlling Additional Variables
	(1)	(2)	(3)
DF	−0.004 ***	0.034 ***	0.041 ***
	(0.001)	(0.011)	(0.013)
Mobile phone penetration rate			0.009
			(0.010)
Constant	1.510	−21.577	−22.416
	(1.434)	(18.775)	(18.726)
Controls	√	√	√
Individual FE	√	√	√
City FE	√	√	√
Year FE	√	√	√
Observations	10,777	11,150	11,150
Adj_R2	0.705	0.572	0.572

*** *p* < 0.01. Robust standard errors are in parentheses.

**Table 5:** Robustness test for excluded sample.

Variables	Excluding the First-Tier Cities	Excluding Hangzhou City	Excluding the Provinces with High DFI Index
	(1)	(2)	(4)
DF	0.036 ***	0.041 ***	0.044 ***
	(0.013)	(0.013)	(0.015)
Constant	−22.993	−21.998	−14.421
	(18.780)	(18.759)	(18.450)
Controls	√	√	√
Individual FE	√	√	√
City FE	√	√	√
Year FE	√	√	√
Observations	10,938	11,117	9849
Adj_R2	0.573	0.572	0.580

*** *p* < 0.01. Robust standard errors are in parentheses.

**Table 6:** IV method results.

	Average DFI Index of Other Cities	Number of Households with Internet Access Last Year	Interaction Term
	(1)	(2)	(3)
DF	0.044 ***	0.182 ***	0.136 **
	(0.014)	(0.058)	(0.063)
Controls	√	√	√
Individual FE	√	√	√
City FE	√	√	√
Year FE	√	√	√
Observations	11,150	10,896	9162
Adj_R2	−0.704	−0.747	−0.733
Cragg–Donald Wald F	32,632.850	303.822	272.848
F statistics of first stage	2093.840	181.950	178.400

*** *p* < 0.01, ** *p* < 0.05. Robust standard errors are in parentheses.

**Table 7:** Heterogeneity results of DF dimensions.

Variables	Explained Variable: Physical Health of the Elderly		
	(1)	(2)	(3)
Coverage breadth	0.002		
	(0.011)		
Usage depth		0.016 **	
		(0.008)	
Digitization degree			0.014 ***
			(0.004)
Constant	−21.409	−22.639	−19.251
	(18.720)	(18.667)	(18.881)
Controls	√	√	√
Individual FE	√	√	√
City FE	√	√	√
Year FE	√	√	√
Observations	11,150	11,150	11,150
Adj_R2	0.572	0.572	0.573

*** *p* < 0.01, ** *p* < 0.05. Robust standard errors are in parentheses.

**Table 8:** Heterogeneity results of intergenerational links.

Variables	Cohabitation with Children	Not Cohabitation but in the Same City	Not Cohabitation and Not in the Same City	Not Cohabitation but in Weekly Contact	Not Cohabitation and No Weekly Contact
	(1)	(2)	(3)	(4)	(5)
DIF	0.050 *	0.068 ***	0.048	0.063 **	0.029
	(0.027)	(0.026)	(0.047)	(0.029)	(0.035)
Constant	−8.598	64.144 *	42.802	74.538 *	10.682
	(29.076)	(33.139)	(52.390)	(42.136)	(42.630)
Controls	√	√	√	√	√
Individual FE	√	√	√	√	√
City FE	√	√	√	√	√
Year FE	√	√	√	√	√
Observations	3192	2601	829	1974	1425
Adj_R2	0.574	0.599	0.518	0.475	0.635

*** *p* < 0.01, ** *p* < 0.05, and * *p* < 0.10. Robust standard errors are in parentheses.

**Table 9:** Heterogeneity results of educational level and urban-rural.

Variables	Low Level of Education	High Level of Education	Rural	Urban
	(1)	(2)	(3)	(4)
DF	0.031	0.049 ***	0.022	0.057 ***
	(0.019)	(0.017)	(0.017)	(0.020)
Constant	−28.035	−1.892	−13.901	−35.466
	(23.223)	(27.300)	(23.416)	(31.590)
Controls	√	√	√	√
Individual FE	√	√	√	√
City FE	√	√	√	√
Year FE	√	√	√	√
Observations	6077	5073	6881	4269
Adj_R2	0.518	0.630	0.564	0.579

*** *p* < 0.01. Robust standard errors are in parentheses.

**Table 10:** Heterogeneity of results per region.

Variables	Eastern	Central	Western
	(1)	(2)	(3)
DF	0.033	0.058 ***	0.033
	(0.027)	(0.020)	(0.032)
Constant	−38.237	−24.905	8.000
	(35.931)	(29.434)	(31.040)
Controls	√	√	√
Individual FE	√	√	√
City FE	√	√	√
Year FE	√	√	√
Observations	3488	4227	3435
Adj_R2	0.543	0.586	0.582

*** *p* < 0.01. Robust standard errors are in parentheses.

**Table 11:** Underlying mechanism results.

Variables	Household Disposable Income	Liquidity Constraints	Medical Service Utilization
	(1)	(2)	(3)
DF	0.013 ***	0.036 *	0.007 *
	(0.004)	(0.022)	(0.004)
Constant	6.249	−47.721 **	1.435
	(5.170)	(19.054)	(4.520)
Controls	√	√	√
Individual FE	√	√	√
City FE	√	√	√
Year FE	√	√	√
Observations	6374	7733	10,257
Adj_R2	0.595	0.534	0.133

*** *p* < 0.01, ** *p* < 0.05, and * *p* < 0.10. Robust standard errors are in parentheses.

## Data Availability

The original data are available at https://charls.pku.edu.cn/ (accessed on 22 May 2023). All the data used in the present study are available upon direct request by contacting the corresponding author.
